# Stromal Vascular Fraction and Amniotic Epithelial Cells: Preclinical and Clinical Relevance in Musculoskeletal Regenerative Medicine

**DOI:** 10.1155/2021/6632052

**Published:** 2021-02-18

**Authors:** Francesca Veronesi, Melania Maglio, Deyanira Contartese, Lucia Martini, Aurelio Muttini, Milena Fini

**Affiliations:** ^1^IRCCS Istituto Ortopedico Rizzoli, Complex Structure of Surgical Sciences and Technologies, Via di Barbiano 1/10, 40136 Bologna, Italy; ^2^Faculty of Bioscience and Agro-Food and Environmental Technology, University of Teramo, Italy

## Abstract

Musculoskeletal regenerative medicine is mainly based on the use of cell therapy to heal damaged tissues such as bone, cartilage, and tendons. Throughout the years, different cell types have been employed for the treatment of musculoskeletal diseases, in particular, mesenchymal stem cells (MSCs) derived from bone marrow (BMSCs) and adipose tissue (ADSCs). Though the results of these literature studies have been encouraging, there are some limitations, especially on long-term results. Recently, some interest has shifted towards new cell types such as the stromal vascular fraction (SVF) and amniotic endothelial cells (AECs). The aim of the present literature review is to evaluate preclinical and clinical studies that used SVF and AECs for musculoskeletal tissue regeneration. Forty-eight preclinical and clinical studies, performed in the last 10 years, were identified. Both SVF and AECs, injected or implanted with or without scaffolds, were shown to be valid alternatives, and in some ways superior, to ADSCs and BMSCs, being able to differentiate towards osteogenic, chondrogenic, and tenogenic lineages, and to promote cell and tissue regenerative potential. The use of SVF and AECs could represent a new regenerative treatment in several musculoskeletal pathologies, solving the problem of cell expansion *in vitro*.

## 1. Introduction

In several musculoskeletal tissue diseases that affect the cartilage, tendons, and bone, there is the need for new regenerative treatments instead of traditional conservative or surgical therapeutic approaches that mainly give palliative care or short-term curative effects [1–3]. In this regard, mesenchymal stem cells (MSCs) have been employed as substitutes and as a promising therapeutic strategy to restore tissue biology, having success in several pathologies, even if the ideal source of stem cells is still debated. In the ambit of cellular therapies, MSCs play a leading role because they possess paracrine activity, through which they exert anti-inflammatory, antiapoptotic, antifibrotic, proangiogenic, and mitogenic activities on the microenvironment, adjacent tissues, and cells [4]. Different tissues have been identified as a source of MSCs, and among them, MSCs from bone marrow (BMSCs) have been mostly used for the regeneration of musculoskeletal tissues, achieving satisfactory results [5]. However, MSCs from adipose tissue (ADSCs) show advantages over BMSCs: ADSCs are reported to have higher genetic stability and higher proliferation, differentiation, and immunoregulatory abilities, and they also show lower senescence than BMSCs [6, 7]. The clinical use of MSCs could be complicated due to donor site morbidity, ageing or disease of the donor, and the necessity of a previous *in vitro* expansion to obtain a large cell number able to produce a clinical effect (it is estimated that nearly 10 × 10^7^ cells should produce a clinically appreciable effect) with associated risks of cell transformation or infection, replaced by a one-step procedure with bone marrow [8].

With particular reference to therapies aimed at skeletal muscle regeneration, other cellular sources have also been tested, including mesoangioblasts, derived from blood vessels, or fibro/adipogenic progenitors (FAPs), multipotent mesenchymal cells derived from skeletal muscle. Mesoangioblasts are multipotent mesodermal progenitor cells that can be isolated by fetal muscle biopsy [9]. FAPs are involved in a dynamic crosstalk with the other cellular populations of the muscle stem cell niche, in particular, immediately after injury occurrence [10]. However, isolation of FAPs requires the application of a long and complex protocol, including muscle dissection or digestion, and subsequent characterization with antibody staining and cell sorting [11].

In an effort to find a smarter cell substitute for MSCs, the stromal vascular fraction (SVF) has been characterized to be employed in preclinical and clinical scenarios [12]. SVF includes not only ADSCs but also a heterogeneous group of cells, such as progenitor cells, endothelial cells, fibroblasts, monocytes, macrophages, immune cells, muscle cells, pericytes, CD34+ cells, growth factors (GFs), a few adipocytes, and stromal components [13]. Similar to MSCs, SVF is proangiogenic and immunomodulatory, and its cellular components are able to differentiate and proliferate, all of the features that make it suitable for tissue regeneration [14]. The advantage of using SVF with respect to expanded ADSCs is immediately clear since SVF, obtained with collagenase digestion and centrifugation of adipose tissue, can be easily harvested from a patient through lipoaspiration. Moreover, it is autologous, requires minimal manipulation, and contains ADSCs at a percentage ranging from 0.06 to 4 CFU-f. Therefore, SVF could be injected directly into a damaged tissue reducing inflammation and promoting regeneration, with consequent reduction in health costs and hours of hospitalization [15, 16]. Indeed, SVF allows the so-called one-step surgical procedure, through which it is possible to harvest and implant SVF in the same surgical session, not requiring *in vitro* expansion. This procedure consists of minimal cell manipulation and low risks linked to culture, without specific regulatory requirements for clinical translation, thus accelerating surgery. The process that goes from surgical adipose tissue harvest, SVF production, and its seeding onto a scaffold or onto hydrogels or its direct injection, lasts at most 4 hours [17, 18].

Another innovative cell source was found in the human placenta, a waste material with cells characterized by high plasticity [19]. The amniotic membrane is obtained from the placenta without an invasive procedure and could be employed as an autologous or allogenic graft due to its immunomodulation properties [20, 21]. The amniotic endothelial cells (AECs) are considered a valid alternative to MSCs because they differentiate into three lineages (osteogenic, adipogenic, and chondrogenic), express mesenchymal and embryonic stem cell markers, show a nontumorigenic phenotype, and have a high yield in terms of in vitro expansion. In addition, AECs replace embryonic stem cells that show a clear impact on ethical matters [22].

Currently, while BMSCs are widely analysed in musculoskeletal pathologies, little is known on the use of SVF and AECs as cell therapies for the regeneration of musculoskeletal diseases especially in comparison with other common cell types and sources [23–26]. These cells were mostly characterized *in vitro* and sporadically compared with other cell types, despite exhibiting noninferior characteristics [27]. However, considering the advantages of the fact that the source of these cells is waste material, which does not include any kind of sampling, it would be interesting to compare them with SVF, whose use in regenerative medicine is very promising, though require a more demanding harvesting. The aim of this review is to collect preclinical and clinical studies, performed in the last 10 years, which used SVF or AECs in bone, cartilage, and tendon tissue regeneration.

## 2. Materials and Methods

The review has been performed according to the Preferred Reporting Items for Systematic Reviews and Meta-Analyses (PRISMA) statement. The studies included in the present review were identified through the http://www.pubmed.com/ and http://www.webofknowledge.com/ databases. In the first database, the keywords used were the following: “(amniotic epithelial stem cells OR stromal vascular fraction) AND (tendon OR cartilage OR bone OR ligament).” The limits were use of English language and publication date from 2010/01/01 to 2019/06/31. In the second database, the keywords were the same, but the limits were use of English language, publication date from 2010 to 2019, and document type was article.

A total of 284 articles were found using the http://www.pubmed.com/ database, and from among them, 242 articles were excluded because they were reviews; or they were focused on cell isolation techniques; or they were not related to musculoskeletal tissues but were related to the heart, corneal epithelium, liver, skin, or bladder; or they were concerned with culture-expanded ADSCs, BMSCs, embryonic stem cells, or MSCs derived from amniotic fluid. Therefore, forty-two studies were accepted.

A total of 452 articles were found using http://www.webofknowledge.com/, and of these, 449 were excluded because they were not inherent, or they were reviews, or they overlapped with the previous search (38 studies). Therefore, three studies were accepted.

**Figure 1:**
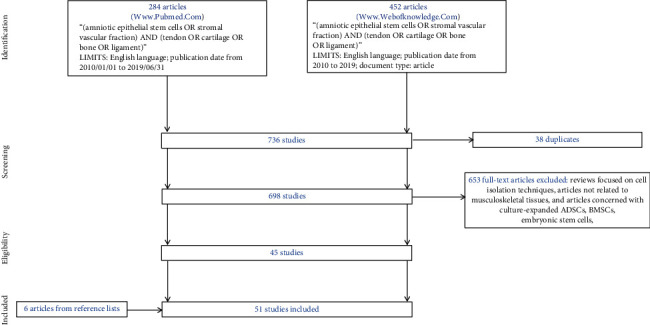
Schematic representation of the search strategy.

In addition, after reading the reference lists of the accepted studies, another six articles were included. Therefore, a final total of 51 studies were taken into consideration (Figure 1).

## 3. Results

**Figure 2:**
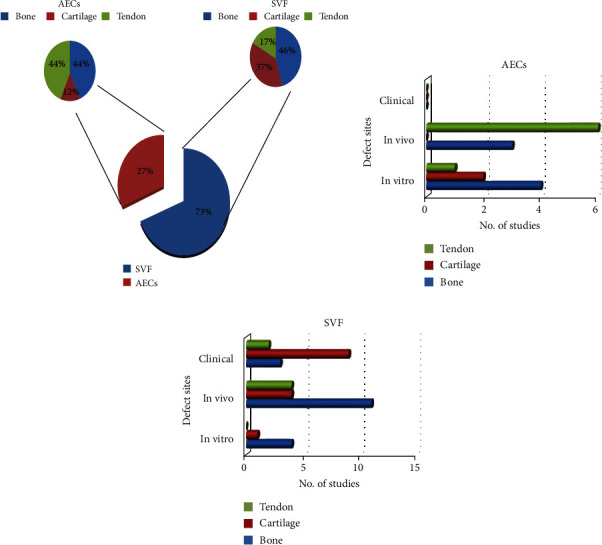
Amount of in vitro, in vivo, and clinical studies. (a) Pie chart of the percentages of the literature studies on SVF (blue: 73%) and AECs (red: 27%). In addition, the other two pie charts for each type of cell present the percentages of literature studies on bone (blue), cartilage (red), and tendon (green) regeneration. (b) Bar graph on the number of clinical, in vivo, and in vitro studies found in the literature, for the regeneration of bone (blue), cartilage (red), and tendon (green) by AECs. (c) Bar graph on the number of clinical, in vivo, and in vitro studies found in the literature, for the regeneration of bone (blue), cartilage (red), and tendon (green) by SVF.

Most of the studies (35/51 studies) dealt with SVF [7, 14, 25–57], and 16/51 studies dealt with AECs [58–73] (Figure 2).

The 35 studies on SVF were conducted to treat bone (no of studies: 16) [17, 28–42], cartilage (no of studies: 13) [7, 43–54], and tendon (no of studies: 6) [55–60] defects (Figure 2). They were conducted *in vitro* [17, 28–30, 43] and *in vivo* [7, 29, 31–39, 42–45, 55–58], and some of them were clinical studies [40–43, 46–54, 59, 60].

Regarding AECs, seven studies were performed on bone [61–67], 2 on cartilage [68, 69], and 7 on tendon [70–76] defects. Among these 13 studies, some were carried out *in vitro* [61–64, 68–70] and others *in vivo* [65–67, 71–76] (Figure 2).

In addition, 11 studies compared two or more cell types in the same study [7, 17, 28, 29, 35, 43, 44, 56, 61, 67, 68]. More precisely, SVF were compared with the following: (1) BMSCs [28, 56], *in vitro* [28] and *in vivo* in rabbit tendon regeneration [56]; (2) ADSCs [7, 17, 35, 44], *in vitro* [17] and *in vivo* in mouse bone defect [35], goat osteochondral defects [44], and sheep knee osteoarthritis (OA) [7]; and (3) monocyte cell line (THP1) and ADSCs [29] or chondrocytes and ADSCs [43] in rat bone defects [29] or mouse subcutaneous pouches [43].

AECs were directly compared with the following: (1) ADSCs *in vitro* [61]; (2) BMSCs and amniotic fluid MSCs (AFMSCs) in mouse subcutaneous pouches [67]; and (3) chondrocytes, BMSCs, and amniotic MSCs (AMSCs) *in vitro* [68].

### 3.1. SVF and Bone

#### 3.1.1. In Vitro Studies

Four *in vitro* studies were performed with human SVF (hSVF) obtained from subcutaneous tissue [29] or nonspecified sites [17, 28, 30] of donors (Table 1).

In the first study, ALP activity and gene expression of Runt-related transcription factor 2 (RUNX2), collagen I (COLL I), alkaline phosphatase (ALP), and Osterix (OSX) of human BMSCs (hBMSCs) significantly increased when cocultured with hSVF in comparison to hBMSCs cultured alone [28].

In another study, ALP activity and calcium content of THP1 increased more when cocultured with hSVF than with hADSCs, after two and four weeks of culture. Additionally, in this study, hSVF or hADSCs alone or combined with THP1 cells were implanted in bone defects in femoral condyles of 46 nude rats. Ten weeks after implanting, it was observed that hSVF increased significantly more bone area (BA) than hADSCs [29].

Two types of scaffolds, namely, poly(L-lactide-co-caprolactone) (PLCL) and COLL I/COLL III, were seeded with hSVF and cultured in normal (NM), chondrogenic (CM), or osteogenic (OM) media to observe hSVF differentiation. The expression of sex-determining region Y box 6 (SOX6) and SOX9 significantly increased when the PLCL construct was cultured in CM. Gene expression of ALP, osteonectin, and COLL I increased when it was cultured in OM. Aggrecan gene expression increased when hSVF was cultured on PLCL, while COLL II gene expression increased with the COLL I/COLL III scaffold [30].

Finally, in another study, hSVF was cultured on a xenohybrid bone graft and compared with hADSCs. hSVF induced the formation of more bone trabeculae than hADSCs after 2 months of culture [14].

#### 3.1.2. In Vivo Studies

Four *in vivo* studies were conducted in subcutaneous or muscular pouches of nude rats [31], athymic mice [32], nude mice [33], and syngenic mice [34]. SVF was obtained from the abdomen or breast [32], epididymis [34], or nonspecified sites [31, 33] of human donors (Table 1).

In one study, a hydroxyapatite (HA) scaffold was implanted alone or combined with hSVF. The addition of hSVF increased vessel number and M2 macrophages after 1 week and bone volume (BV) after 2 months [31].

In another study, beta-tricalcium phosphate (*β*TCP) or HA scaffolds were seeded with hSVF at 3 different concentrations (<2 × 10^6^ CFU-f/cm^3^, >2 × 10^6^ CFU-f/cm^3^, or >3 × 10^6^ CFU-f/cm^3^). After 2 months from implantation, both scaffolds, seeded with hSVF at concentrations of >2 × 10^6^ CFU-f/cm^3^ or >3 × 10^6^ CFU-f/cm^3^, increased the formation of dense matrix [32]. An increase in mineralized volume, BA, osteoid matrix formation, and vessel length was observed 3 months after the implantation of a devitalized hypertrophic cartilage pellet with hSVF at a concentration of 6 × 10^6^/ml or 12 × 10^6^/ml in comparison to a concentration of 24 × 10^6^/ml. Moreover, BA was significantly higher in nude rats with bilateral parietal bone defects treated with a scaffold and hSVF than with a scaffold alone after 1 month [33].

Najman et al. implanted a deproteinized sterilized bovine bone scaffold alone or combined with autologous SVF and platelet-rich plasma (PRP). Evaluations were performed after 1, 2, 4, and 8 weeks when it was observed that SVF and PRP significantly increased osteopontin (OPN) protein levels; gene expression of OSX, COLL I, ALP, and osteocalcin (OCN); osteoid-like tissue formation; and vascularization compared to a scaffold alone [34].

The other 5 *in vivo* studies were performed in critically sized calvaria defects in nude mice [35] or in rats [35], in segmental bone defects in the middiaphysis of the left ulna of rabbits [37], in osteonecrosis jaw- (ONJ-) like defects of mice [38], and in the right carpal bone of 1 horse [37]. SVF was obtained from the flank, scapula, abdomen, inner thigh [35], inguinal region [36], and gluteal muscle [39], and used as autologous [36, 39], allogenic [37, 38], or xenogenic grafts [32] (Table 1).

Both hSVF and hADSCs, seeded onto a polycaprolactone-decellularized bovine bone extracellular matrix (PCL-DCB) scaffold, increased BV more than the scaffold alone; in addition, hSVF increased BV during time, up to 3 months, in critically sized defects. In the same study, the PCL-DCB scaffold, seeded with hSVF and hADSCs, was also implanted in muscular pouches of nude mice, showing a high vascular volume in the presence of hSVF up to 6 weeks later [35]. Demineralized bone matrix (DBM) or DBM combined with polylactic acid (PLA), seeded or not with SVF, were always used in critically sized defects, and after 2 months, it was observed that when used alone, both scaffolds increased defect filling, but when combined with SVF, they also increased BA and OCN protein levels [36].

The poly(lactide-co-glycolide) (PLGA) scaffold was seeded with SVF, osteogenically induced or not, and implanted into rabbit ulna defects. Two months after implanting, SVF that was not osteogenically induced increased bone volume fraction (BVF), while SVF that was osteogenically induced also increased defect bridging, trabecular bone, and trabecular thickness (Tb.Th) [37].

In ONJ-like lesions, after 2 and 4 weeks, SVF increased living BA, osteocytes, bone filling, Tb.Th, bone mineral density (BMD), collagen fibers, blood vessels, and F4/80+ macrophages, while it reduced necrotic BA, empty lacunae, trabecular separation (Tb.Sp), and polymorphonuclear (PMN) infiltration in comparison with saline solution [38].

Finally, SVF injection in the carpal bone allowed a horse to return to racing after 4 months with no injuries and lameness and even with a higher performance level after 20 months [39].

#### 3.1.3. Clinical Studies

Three clinical studies were performed in 10 edentulous patients [40, 41] and in 8 patients with displaced low-energy fractures of the proximal humerus [42]. SVF was autologous and was obtained from the abdomen [41, 42] or nonspecified sites [40] (Table 1).

In two studies, biphasic calcium phosphate (BCP) or *β*TCP scaffolds, with or without SVF, were implanted in edentulous patients [40, 43]. After 6 months, the first study showed similar bone formation and blood vessels in all treatments [40], while the second study showed higher BV and osteoid volume (OV) when SVF was added to scaffolds in comparison to the same scaffolds alone, with no adverse effects after 3 years [41].

Finally, through the setup of a clinical trial, it was shown that the SVF pellet, mixed with fibrin hydrogel and silicate HA microgranules, reduced pain and increased bone ossicles 1 year after implantation in low-energy fractures of the humerus. In the same study, a previous *in vivo* study was performed in a critically sized segmental defect of the femora in nude mice, in which porous silicate HA microgranules and hSVF increased mineralized volume, BV, and early stage of maturation, 2 months after implantation [42].

### 3.2. SVF and Cartilage

#### 3.2.1. In Vitro Studies

One *in vitro* study was performed with hSVF or hADSCs harvested from the abdomen, and with human chondrocytes (Table 2). After 1 month, the addition of hSVF to chondrocyte cultures induced a higher amount of glycosaminoglycans (GAG) and increased cell proliferation than hADSCs cocultured with chondrocytes. In the same study, allogenic chondrocytes combined with allogenic SVF increased GAG and COLL II 2 months after they had been implanted in subcutaneous pouches of nude mice [43].

#### 3.2.2. In Vivo Studies

Three *in vivo* studies were conducted in goats with osteochondral defects in the trochlea femoris and the medial femoral condyle [44], in sheep with knee OA [7], and in NOD/SCID mice with cartilage injury [45]. SVF was derived from nonspecified sites [44], the cardiothoracic region [7], or the abdomen [45] and was autologous [7, 44] or xenogenic [45] (Table 2).

In the first study, SVF and ADSCs were seeded onto COLL I/COLL III scaffolds and implanted into osteochondral defects. After 4 months, scaffolds with SVF induced higher hyaline cartilage and subchondral bone (SB) regeneration than a scaffold alone or a scaffold combined with ADSCs [44].

In the second study, in the presence of OA, SVF, combined with hyaluronic acid, reduced COLL X and increased stromal cell-derived factor 1 (SDF1) protein production in comparison to ADSCs or hyaluronic acid alone [7].

In the third study, hSVF mixed with PRP significantly reduced the recovery time (in terms of animal movement) and cartilage lesion area more than phosphate-buffered saline (PBS), 45 days from injection into cartilage injuries [45].

#### 3.2.3. Clinical Studies

Eight clinical studies were performed in patients with OA, full-thickness chondral defect of the talar dome [50], and traumatic osteochondral defect of the right femoral condyle [54].

Allogenic or autologous SVF was obtained from the abdomen [42–44, 46–48, 50], periumbilical region [45], and buttocks [49] (Table 2).

In patients with OA, the treatments consisted of SVF alone [46, 47]; SVF and PRP [48, 49]; SVF, PRP, and arthroscopic microfracture [50, 51]; or SVF and arthroscopic debridement [52].

The injection of SVF alone reduced changes in Japanese Knee Osteoarthritis Measure (JKOM), Western Ontario and McMaster Universities Osteoarthritis Index (WOMAC), and visual analog scale (VAS) scores after 1, 3, 6, and 18 months [46, 47] with no infection, thromboembolism, or adverse reactions [47]. The combination of SVF and PRP reduced WOMAC scores and increased the six-minute distance parameter after 3 months, 6 months, 1 year, and 2 years [48] and ameliorated Knee injury and Osteoarthritis Outcome Score (KOOS), symptoms, and functional activity at 1 year [49]. Arthroscopic microfracture was accompanied with SVF injection with or without PRP. With the addition of PRP, bone marrow edema and WOMAC score was reduced and Lysholm score was increased after 12 and 18 months [50], while without PRP, bone marrow edema, VAS, WOMAC, and Outerbridge scores were reduced after 12 and 24 months [51]. In one study, in which OA was treated with arthroscopic debridement, the addition of SVF reduced VAS and WOMAC scores and increased the range of motion (ROM) more than hyaluronic acid at 1, 3, 6, and 12 months, while it increased the Magnetic Resonance Observation of Cartilage Repair (MOCART) score and complete tissue filling more than hyaluronic acid at 6 and 12 months [52].

Regarding the full-thickness chondral defect, SVF, associated with arthroscopic marrow stimulation, reduced the VAS score and increased the American Orthopedic Foot and Ankle Score (AOFAS) and the Tegner and MOCART scores more than the technique alone after 25 months [53].

In a traumatic osteochondral lesion, microfracture associated with SVF and fibrin sealant increased the International Knee Documentation Committee (IKDC) score, the EuroQol-visual analog scale (EQ-VAS) score, and the recovery of the cartilage thickness and reduced bone edema after 1 and 2 years [54].

### 3.3. SVF and Tendon

#### 3.3.1. In Vivo Studies

Four *in vivo* studies were performed in rabbits [55–58] with transection of the midsubstance of the deep digital flexor (DDF) tendon [55], transection of the central one third of the flexor tendon [56], or with the supraspinatus tendon severed from the great trochanter [57, 58]. SVF was harvested from the inguinal region and was allogenic [55, 56] or autologous [57, 58] (Table 3).

SVF was injected in the complete transection of the DDF tendon, and after 2 months, fibrillar linearity and continuity, COLL I production, ultimate load, energy absorption, and stiffness increased, while the number of capillaries and COLL III production decreased [55].

SVF and BMSCs, injected in the complete transection of the flexor tendon, increased energy absorption, ultimate load, ultimate stress, yield load, and stiffness after 3 and 8 weeks [56].

After 1, 2, and 3 months from SVF injection into the supraspinatus tendon severed from the great trochanter, maximum load, maximum strength, stiffness, and signal-to-noise quotient (SNQ) increased [57], as well as tendon-bone healing, COLL I, and bone morphogenetic protein 2 (BMP2) after 2 months [58].

#### 3.3.2. Clinical Studies

The two clinical studies were performed. One study included 45 patients with noninsertional Achilles tendinopathy (NIAT) [59], and the other study included 44 patients with chronic tendinopathy of the Achilles tendon [60]. SVF was allogenic and harvested from the abdomen [59, 60] (Table 3).

In the first study, both PRP and SVF reduced VAS and increased MR size, US size, and peri- and intratendinous flow (PD), while only SVF increased MR signal intensity (MR-Si) after 6 months [59].

Similarly, PRP and SVF were compared in Achilles tendon tendinopathy. Both PRP and SVF reduced VAS and increased the Victorian Institute of Sports Assessment—Achilles (VISA-A) questionnaire score, AOFAS score, and SF-36 score after 2 weeks, 1, 2, 4, and 6 months, showing better results with SVF [60].

### 3.4. AECs and Bone

#### 3.4.1. In Vitro Studies

Four *in vitro* studies were conducted with human AECs (hAECs) [61–64] (Table 4).

In the first study, hAECs and hADSCs were compared in terms of osteogenic and chondrogenic differentiation. After 3, 7, and 14 days of culture, hAECs showed higher RUNX2 and SOX9 gene expression, OCN, aggrecan, and COLL II protein than hADSCs. After 14 and 28 days, mineralization was more pronounced in hAECs than in hADSCs [61].

Keeping in terms of osteogenic differentiation, hAECs, cultured in OM and stimulated with pulsed electromagnetic fields (PEMFs), increased ALP, BMP2, RUNX2, nuclear factor erythroid 2-related factor 2 (NRF2), Kelch-like ECH-associated protein 1 (KEAP1) and OCN gene expression, ALP activity, and OCN protein and calcium deposition in comparison to cells in NM or in OM, without PEMFs, after 3, 7, 11, and 21 days. The addition of PEMFs in NM also induced ALP and OCN gene expression, ALP activity, and OCN protein and calcium deposition, after 3, 7, 11, and 21 days [62].

The effect of conditioned medium from hAECs, produced after 24 hours of culture, on human fetal osteoblast cell line (hFOB1.19) was evaluated in one study. Conditioned medium increased hFOB1.19 cell migration and proliferation; ALP activity; and ALP, OCN, OPN, and RUNX2 gene expression after 2 hours and 1, 2, 3, and 6 days. The addition of the antibody against transforming growth factor *β* (TGF*β*1) in the culture medium reduced ALP activity, ALP and OCN gene expression, and cell migration after 6 hours and 6 days [63].

Finally, a study showed how mechanical stretch (with a maximum uniaxial stretched length of 7.35 cm, 5% of elongation, and frequency of 0.5 Hz) increased OCN, RUNX2, ALP, *β-*catenin, and Cyclin D1 gene expression and protein expression 6, 12, and 24 hrs after stimulation, with a synergic effect when OM was added [64].

#### 3.4.2. In Vivo Studies

Three studies were conducted in sheep submitted to sinus augmentation [65], in rats with maxillary alveolar defect [66], and in subcutaneous pouches of nude mice [67]. AECs were allogenic [65] or xenogenic [66, 67] (Table 4).

Ovine AECs, seeded onto a calcium phosphate (CaP) scaffold and implanted into alveolar defects, increased vascular endothelial growth factor (VEGF), vascular area, and new bone more than the scaffold alone after 3 months [65]. Similarly, a *β*TCP scaffold, seeded with hAECs, reduced Tb.Sp and CD68 cells and increased BMD, BV, trabecular number (Tb.N), BA, and VEGF more than the scaffold alone after 1 and 2 months [66].

Three types of cells, namely, hAECs, hBMSCs, and hAFMSCs, were seeded onto the *β*TCP scaffold and implanted into subcutaneous pouches, showing higher viable cells and higher OPN and OCN protein production than the scaffold alone, regardless of cells, after 1 month [67].

### 3.5. AECs and Cartilage

#### 3.5.1. In Vitro Studies

There were two *in vitro* studies conducted with hAECs [68, 69] (Table 5).

In the first study, cartilage samples, with a defect of 2 mm, were *ex vivo* treated with a human amniotic membrane (HAM) scaffold seeded with human chondrocytes, hBMSCs, hAECs, or hAMSCs and added with the respective cell pellets. After 2 months of culture, all treatments showed the same degree of repair and International Cartilage Repair Society (ICRS) score [68].

In the second study, micromasses of hAECs were cultured with TGF*β*1 or BMP7 for 3 days and 3 weeks. The addition of BMP7 increased SOX9 and COLL II gene expression at 3 days, while TGF*β*1 increased it to 3 weeks. Both treatments increased proteoglycan gene expression during time [69].

### 3.6. AECs and Tendon

#### 3.6.1. In Vitro Studies

One *in vitro* study was conducted with ovine AECs alone or cocultured with fetal or adult ovine tenocytes or tendons (Table 6). After 7, 14, and 28 days of culture, fetal tendons increased AEC migration, while adult tenocytes or tendons reduced AEC proliferation. Fetal tenocytes or tendons increased AEC proliferation and OCN, tenomodulin (TNMD), and scleraxis (SCX) protein production. In addition, fetal tendons reduced the telomere area (TEA) and feret maximum (TEF) and mean densitometric (MEAND) values, while adult tendons or tenocytes reduced COLL III gene expression and TNMD protein [70].

#### 3.6.2. In Vivo Studies

Six *in vivo* studies were conducted in horses [71, 72] with monolateral acute superficial digital flexor tendon (SDFT) injuries [71] and acute or chronic SDFT injury in the midmetacarpal region [72] or in sheep [73–76] with a bilateral full-thickness hole of flexor digitorum superficialis tendon (FDST) [73, 75] or bilateral defect of the middle portion of the Achilles tendon [74, 76]. AECs were xenogenic [71, 72, 76] or allogenic [73–75] (Table 6).

A reduction in COLL III and an increase in COLL I and Ki-67 proteins after 2 months from ovine AEC injection into SDFT injuries were observed [71].

Ovine AECs induced a cross-sectional area similar to the healthy tendon, parallel collagen fibers, and no vascularization in SDFT tendinopathy after 6 months [72].

Ovine AECs, injected into the hole of FDST, induced greater improvement in tendon microarchitecture, proliferation index, COLL I gene expression, maximum failure load, and stiffness, as well as greater reduction in vascular area (VA); leukocyte infiltration; macrophage infiltration; and CD86, IL12b, and COLL III gene expression than fibrin glue (FG) after 7, 14, and 28 days [73, 75].

Finally, 1 month after ovine AEC injection, an increase in collagen fibers and no inflammation were observed in defects of the Achilles tendon [74], with a rapid recovery; higher biomechanics; higher TNMD, THBS4, CD206, and IL10 gene expression; and lower CD86 and IL12b gene expression in comparison to FG [76].

## 4. Discussion

Treatments for the regeneration of musculoskeletal tissue disorders of bone, cartilage, and tendon could be conservative or surgical. Rehabilitation, anti-inflammatory drugs, biophysical therapy, platelet derivatives (i.e., PRP) or cell therapy [1, 77], microfracture, arthroscopy [1], and the implantation of grafts or scaffolds or hydrogels [78] are the main treatment options alone or in combination. Moreover, due to the lesion type and site or the patient characteristics (i.e., age, comorbidities, and lifestyles), some treatments reportedly do not have the ability to regenerate the original healthy tissue or have a short-term or incomplete effect.

Preclinical research has always been focused on finding increasingly innovative and less invasive therapies. In this regard, MSCs, in particular ADSCs and BMSCs, but also MABs and FAPs, have been used in musculoskeletal tissue regeneration, even if they show some limitations: the necessity of two surgical steps (one for the harvesting and one for the implantation after *in vitro* expansion), patient morbidity, and the necessity to obtain a huge number of cells through *in vitro* expansion with associated risks.

Nowadays, a novel source of MSCs and new techniques to isolate such cells are being studied. Both SVF and AECs are spreading as new protagonists in the field of cell-based therapies for regenerative medicine, starting from the first applications in cardiology, and it is interesting to note that the only study in which both were used was aimed at cardiac repair [79].

In the field of regenerative medicine, the use of SVF has a relatively recent history. One of the pivotal studies in the introduction of the employment of SVF for clinical purposes dates to 2001, when Zuk et al. described the characterization of multilineage cells harvested from human adipose tissue and called them “processed lipoaspirate.” In this study, it was demonstrated that the collected SVF contained not only adipose cells but also mesodermal or mesenchymal origin cells, as well as pericytes, endothelial cells, and smooth muscle cells. The evidence of the cells' capability to differentiate *in vitro* towards adipogenic, chondrogenic, myogenic, and osteogenic lineages opened the possibility of exploiting this as a new source for tissue regeneration [12]. The protocol described in this paper became a reference to isolate SVF and study its composition; however, until about ten years ago, the investigation was limited to *in vitro* evaluation of SVF derived from mainly rodent models and tested for characterization and regeneration, with particular reference to cardiac muscle [80, 81]. One of the first applications for a musculoskeletal system *in vivo* available in literature compared the efficacy of SVF and BMSCs in the treatment of OA in an equine model, without however obtaining any evident results in favour of neither treatment [82]. Since then, the number of studies has increased, while the application of SVF in clinical trials is fairly recent, as evidenced by this review.

As for AECs, despite the extensive literature history, it was only after the First International Workshop on Placenta-Derived Stem Cells when it was finally pointed out the field of application of mesenchymal stromal cells isolated from various parts of the placenta or epithelial cells isolated from the amniotic membrane in regenerative medicine. The findings suggested that the main applications were oriented mainly towards hepatic and cardiac repair and neurological disorders [83].

This review focuses on preclinical and clinical studies on an innovative source of cells (amniotic membrane) and a one-step surgical technique not based on *in vitro* expanded cells (SVF transplantation) for musculoskeletal tissue regeneration.

In the last 10 years, 48 preclinical and clinical studies employed SVF (73%) [7, 17, 28–60] or AECs (27%) [61–76] in musculoskeletal tissue regeneration.

In general, in the studies of these cell sources, researchers prevalently performed in vivo studies [7, 29, 31–39, 42–45, 56–58, 65–67, 71–76], followed by clinical ones [40–42, 46–54, 59, 60] and in vitro studies [17, 28–30, 43, 61–64, 68–70]. As for AECs, no clinical studies have yet been conducted, and the only two studies that analysed AEC behavior in cartilage were *in vitro* [68, 69]. For both cell types, most of the studies regarded bone regeneration [17, 28–42, 61–67], followed by cartilage [7, 43–54, 68, 69] and tendon [55–60, 70–76]. SVF was prevalently implanted with scaffolds for bone regeneration: HA [31, 32], *β*TCP [32, 40, 41], devitalized hypertrophic cartilage pellet [33], deproteinized sterilized bovine bone [34], PCL-DCB [35], DBM [36], PLA [36], PLGA [37], or fibrin hydrogel added with porous silicate HA microgranules [42]. Conversely, in cartilage regeneration, SVF was prevalently injected without scaffolds [43, 45], especially in the clinical studies [46–54], even if two studies employed COLL I/COLL III [44] or hyaluronic acid [7]. All the studies on tendon employed SVF without scaffolds [55–60].

*In vivo* AECs were seeded onto CaP [65] or *β*TCP [66, 67] scaffolds in bone defects, while in tendon defects the authors injected AECs without scaffolds [70–76].

For both SVF and AECs, the results of the preclinical studies were obtained prevalently with histology and/or histomorphometry [29, 31–38, 42–45, 55, 58, 65–68, 71–76], protein production detection through immunohistochemistry (IHC) [7, 17, 31, 34, 36, 38, 42, 55, 58, 60, 65–68, 71, 75, 76], and micro-CT [17, 31, 33, 35, 37, 38, 44, 65, 66] and RT-PCR for gene expression analysis [23, 30, 34, 61–63, 69, 70, 73, 75, 76].

The preclinical results showed that SVF increased BMSCs' osteogenic differentiation, ALP activity, and the calcium content of THP1 cells [28, 29], and it was able to differentiate towards osteogenic, chondrogenic, or tenogenic lineages both *in vitro* and in heterotopic sites [17, 30–35, 43, 55]. SVF also increased BV, defect filling, Tb.Th, and cartilage or tendon regeneration in animal models of calvaria [35, 36], ulnar [37], ONJ-like [38], lameness [39], osteochondral [44], OA [7], cartilaginous [45], flexor tendon [55, 56], and supraspinatus tendon [57, 58] defects.

AECs have an osteogenic [61, 62, 64, 67], chondrogenic [69], or tenogenic [70] differentiation ability, *in vitro* and in heterotopic sites; increased BA, vessel formation, BMD in sinus defects [65, 66], and regenerated *ex vivo* cartilage defects [69]; and acute or chronic SDFT, FDST, and Achilles [71–74] tendon lesions.

Clinical studies were conducted in edentulous patients [40, 41] or in patients affected by humerus fractures [42], OA [46–52], full-thickness chondral defects [53], osteochondral lesions [54], NIAT [59], and Achilles tendon lesions [60]. The measurements were performed with micro-CT [41, 42], histology and histomorphometry [40–42], clinical scores (WOMAC, KOOS, VAS, Lysholm, JKOM, IKDC, PD, VISA-A, AOFAS and EQ-VAS scores, and ROM) [45–54, 59, 60] and radiography (MOCART score, MR-Si, and MR size) [47, 52, 53, 59].

In these studies, SVF could be employed not only as autologous cells but also as allogenic ones, as observed in some studies in which allogenic SVF was used in rabbits [37, 55, 56], mice [38, 43], and in patients affected by OA [46, 48] or tendinopathy [59, 60]. One of the major potential advantages of AECs is that, unlike other cells, they can be used as allogeneic or xenogenic cells. Allogenic AECs were implanted into sheep [65, 73–75] while xenogenic ones in rats [66], horses [71, 72], and sheep [76]. To obtain an idea on other ongoing clinical trials, a further search was carried out on http://www.clinicaltrial.gov/. From this search, six clinical trials were found using SVF in OA patients, 2 in bone and 1 in tendon defects. However, the absence of clinical trials regarding AECs underlines that much remains to be explored on the potential of these cells.

More precisely, in OA patients, randomized clinical studies, single or triple blind in phase I, II, or III, are evaluating the effects of SVF injections into knees, also comparing SVF with corticosteroids, umbilical cord MSCs, BMSCs, or activated PRP. The enrolled patients are 20, 30, 40, 52, 200, and 480, and the evaluations are performed with clinical scores, ROM, adverse event recording, radiography, and arthroscopic cartilage repair assessment after 1, 3, 6, 9, 12, 18, and 24 months.

In 8 and 5 patients, two clinical trials in phase II have evaluated the effect of SVF, seeded or not onto composite HA microgranules after embedding in a fibrin gel, in the treatment of proximal humeral fractures in osteoporotic patients or in craniofacial injuries. Radiological and clinical scores and average tissue thickness are evaluated at 6 and 12 weeks and 1, 3, 6, 9, and 12 months. [42]

One randomized, quadruple blind, phase II clinical trial has been performed in 52 patients to evaluate the ability of autologous SVF in improving the repair of chronic rotator cuff tears, and the evaluations of clinical measurements, change in muscle stiffness, and change in fatty infiltration are carried out at 6 weeks and at 6, 12, and 24 months [58].

The overview of the state-of-the-art in the study of these cell sources highlights some limitations in the comparison of SVF or AECs with other cell types, which is represented by the paucity of preclinical studies and the insufficient number of clinical trials conducted. However, despite these limits, some considerations about the comparison of AECs and SVF vs. other cell types can be made. For example, a superiority of SVF over ADSCs was found in inducing ALP activity and calcium content in THP1 cells [29], in osteogenic differentiation ability [17, 35], in chondrogenic differentiation [43], and in osteochondral or OA defect regeneration [7, 44]. In addition, SVF regenerated biomechanical properties of flexor tendon defects in a similar manner to BMSCs [56]. On the other hand, AECs showed higher osteogenic differentiation ability than ADSCs [61], but similar osteogenic differentiation to BMSCs and AFMSCs [67] and similar chondral defect regeneration to BMSCs and AMSCs [68].

## 5. Conclusions

SVF and AECs are two promising cells for regenerating bone, cartilage, and tendon. Both show advantages in terms of application in a one-step approach, which has become one of the main goals for streamlining and reducing bias in surgical procedures. In addition, harvesting procedures are easy and less invasive in comparison to other cell sources. In addition, as for AECs, many ethical concerns have been overcome when dealing with waste material; at the same time, the harvesting technique of SVF might make this source exploitable for autologous use also in patients with a particular pathology or undergoing therapies. Several preclinical studies affirm the regenerative ability of both SVF and AECs; less clinical studies on SVF exist, while no studies on AECs exist. Most of the clinical studies deal with SVF in patients affected by OA. Additionally, even if there are only a few studies that compare SVF or AECs with other cell types, they showed that SVF and AECs behave in a better or similar way to *in vitro* culture-expanded ADSCs or BMSCs, without showing the now known limitations linked to the cells expanded in culture. An advantage of AECs, compared to other cells, is its use in the form of allogeneic or xenogenic cells, although, in order to affirm this, clinical studies are necessary.

## Figures and Tables

**Table 1:** *In vitro*, *in vivo*, and clinical studies on SVF in bone regeneration.

Materials	Treatment groups	Evaluations	Results	Ref
*In vitro*: hSVF from 4 donors (42-62 yrs) (5 × 10^4^) Purchased hBMSCs (5 × 10^4^)	Group 1: BMSCs Group 2: BMSCs+SVF	ALP activity RT-PCR (RUNX2, COLL I, ALP, and OSX)	12, 24, and 48 hrs Group 2: ↑ gene expression than group 1 24 and 48 hrs Group 2: ↑ ALP activity than group 1	[28]

*In vitro*: hSVF (3 × 10^6^) or hADSCs (1 × 10^6^) from subcutis of 3 donors (38-52 yrs) THP1 (1 × 10^6^) *In vivo*: 46 nude rats with bilateral bone defects (3 × 3 mm) in femoral condyles hSVF (3 × 10^6^) or hADSCs (1 × 10^6^) from abdomen THP1 (1 × 10^6^)	*In vitro*: Group 1: THP1 Group 2: SVF Group 3: THP1+SVF Group 4: ADSCs Group 5: THP1+ADSCs *In vivo*: Group 1: no treatment Group 2: THP1 Group 3: SVF Group 4: THP1+SVF Group 5: ADSCs Group 6): THP1+ADSCs	*In vitro*: ALP activity Calcium content *In vivo*: Histology Histomorphometry	*In vitro*: 2 wks Group 3: ↑ ALP activity than groups 2, 4, and 5. 1 mo Group 3: ↑ calcium content than groups 2 and 5 *In vivo*: 4 and 10 wks Groups 3, 4, 5, and 6: ↑ BA/TA than groups 1 and 2. 10 wks Groups 3 and 4: ↑ BA/TA than groups 5 and 6	[29]

*In vitro*: hSVF (2 × 10^6^) from 8 donors (39.8 ± 9.7 yrs) PLCL scaffold COLL I/COLL III scaffold	Group 1: PLCL+SVF in NM Group 2: COLL I/COLL III+SVF in NM Group 3: PLCL+SVF in CM Group 4: PLCL+SVF in OM Group 5: COLL I/COLL III+SVF in CM Group 6: COLL I/COLL III+SVF in OM	RT-PCR (ACAN, SOX6, SOX9, ALP, ONC, COLL I, COLL III, and COLL X)	4 days Group 3: ↑ ACAN gene expression than group 5) 2 wks Groups 3 and 5: ↑ SOX6, SOX9 gene expression during time Groups 4 and 6: ↑ ALP, ONC, and COLL I gene expression during time 3 wks Group 5: ↑ COLL II gene expression than group 3 Groups 3 and 5: ↓ COLL X gene expression during time	[30]

*In vitro*: hSVF or hADSCs (1 × 10^6^) from 7 donors Xenohybrid bone graft scaffold (7 × 3 mm)	Group 1: SVF in plastic in NM Group 2: ADSCs in plastic in NM Group 3: scaffold+SVF in NM Group 4: scaffold+ADSC in NM Group 5: SVF in plastic in OM Group 6: ADSCs in plastic in OM Group 7: scaffold+SVF in OM Group 8: scaffold+ADSC in OM	Micro-CT IHC (OCN) ALP activity Mineralization	2 mo Group 7: ↑ bone trabeculae than groups 4, 8, and 3 Groups 3 and 4: ↓ OCN than groups 7 and 8 Group 1: ↑ ALP activity than group 2 Groups 1 and 2: ↓ mineralization than groups 5 and 6	[17]

*In vivo*: 28 nude rats with subcutaneous pouch hSVF (12 × 10^6^) from 5 donors (43 ± 12 yrs) HA scaffold (1 cm diameter, 1 cm height)	Group 1: scaffold Group 2: scaffold+SVF previously cultured for 5 days	Micro-CT Histomorphometry	1 wk Group 2: ↑ vessel number, M2 macrophages than group 1 2 mo Group 2: ↑ BV/TV than group 1	[31]

*In vivo*: CD1^nu/nu^ athymic mice with subcutaneous pouches hSVF from abdomen or breast of donors (32 ± 15 yrs) *β*TCP scaffold (8 mm diameter, 4 mm height) HA scaffold (8 mm diameter, 4 mm height)	Group 1: *β*TCP+SVF (>2 × 10^6^ CFU-f/cm^3^) Group 2: *β*TCP+SVF (<2 × 10^6^ CFU-f/cm^3^) Group 3: *β*TCP+SVF (>3 × 10^6^ CFU-f/cm^3^) Group 4: HA+SVF (>2 × 10^6^ CFU-f/cm^3^) Group 5: HA+SVF (<2 × 10^6^ CFU-f/cm^3^) Group 6: HA+SVF (>3 × 10^6^ CFU-f/cm^3^)	Histology	2 mo Groups 1, 4, 3, and 6: ↑ dense matrix than groups 2 and 5 Groups 3 and 6: ↑ dense matrix similar to osteoid than groups 1 and 4	[32]

*In vivo*: Nude mice with subcutaneous pouches Nude rats with bilateral defects in parietal bone (4 mm) Devitalized hypertrophic cartilage pellet from 5 donors (35.4 ± 11.3 yrs) hSVF from 12 donors (33.7 ± 7.7 yrs)	Group 1: pellet in pouches Group 2: pellet+SVF (6 × 10^6^/ml) in pouches Group 3: pellet+SVF (12 × 10^6^/ml) in pouches Group 4: pellet+SVF (24 × 10^6^/ml) in pouches Group 5: pellet in bone defects Group 6: pellet+SVF (12 × 10^6^/ml) in bone defects	Micro-CT Histology	3 mo Group 3: ↑ mineralized volume, BA than group 1 Groups 2 and 3: ↑ vessel length density than group 1 1 mo Group 6: ↑ BA than group 5	[33]

*In vivo*: 24 syngenic Balb/c mice (8 wks) with subcutaneous pouches Autologous SVF (5 × 10^5^) from epididymis Bio-Oss® scaffold (deproteinized sterilized bovine bone) (10 mg)	Group 1: scaffold Group 2: scaffold+SVF+PRP	Histology IHC (OPN) Histomorphometry RT-PCR (OSX, OCN, COLL I, and ALP)	2 wks Group 2: ↑ OPN protein than group 1 2 and 4 wks Group 2: ↑ OSX, OCN gene expression than group 1 1, 2, and 4 wks Group 2: ↑ COLL I gene expression than group 1 2 mo Group 2: ↑ ALP gene expression, osteoid-like tissue, OPN protein, vascularization than group 1	[34]

*In vivo:* 14 nude immunocompromised mice (8 wks) with bilateral critically sized calvarial defects Nude mice with muscular pouches hSVF or hADSCs (2 × 10^4^) from flank, scapula, abdomen, or inner thigh of donors PCL-DCB scaffold (4 mm diameter, 0.64 mm length)	Group 1: scaffold in bone defect Group 2: scaffold+SVF in bone defect Group 3: scaffold+ADSCs in bone defect Group 4: scaffold in pouches Group 5: scaffold+SVF in pouches Group 6: scaffold+ADSCs in pouches	Micro-CT Histology	3 mo Groups 2 and 3: ↑ BV than group 1 Group 2: ↑ BV during time 10 days Group 5: ↑ VA than groups 4 and 6 6 wks Groups 5 and 6: ↑ VA than group 4	[35]

*In vivo*: 50 SD rats (8 wks) with critically sized calvarial defects Autologous SVF (1 × 10^5^) from inguinal region DBM scaffold PLA scaffold	Group 1: no treatment Group 2: DBM Group 3: DBM+SVF Group 4: DBM+PLA Group 5: DBM+PLA+SVF	Gross evaluations Histology IHC (OCN)	2 mo Groups 3 and 5: ↑ defect filling, BA, OCN than groups 1, 2, and 4 Group 2: ↑ defect filling than groups 1 and 4 Group 4: ↑ defect filling than group 1	[36]

*In vivo*: 15 NZW rabbits (14-16 wks) with unilateral segmental bone defect in the middiaphysis of left ulna (20 mm) Allogenic SVF (1 × 10^6^) from suprascapular region PLGA scaffold (4 × 20 mm)	Group 1: scaffold Group 2: scaffold+SVF Group 3: scaffold+osteogenically differentiated SVF	Radiography Micro-CT Histology	2 mo Group 3: defect bridging, trabecular bone with smooth margins Group 3: ↑ BVF than groups 1 and 2 Group 3: ↑ Tb.Th than group 1 Groups 2 and 3: scaffold absorbed Group 2: ↑ BVF than group 1	[37]

*In vivo*: 28 C57BL/6J mice (8-12 wks old) with bilateral ONJ-like lesions Allogenic SVF (4 × 10^6^) from inguinal region	Group 1: saline Group 2: SVF	Micro-CT Histology Histomorphometry	2 and 4 wks Group 2: ↑ BA, osteocytes, bone filling, Tb.Th, BMD, collagen fibers, blood vessels, VA, F4/80^+^ macrophages; ↓ necrotic BA, empty lacunae, Tb.Sp, PMN infiltration than group 1	[38]

*In vivo*: 1 thoroughbred gelding in training (5 yrs) with lameness of the right carpal bone Autologous SVF (20 × 10^6^) from region above dorsal gluteal muscles	Group 1: SVF	Clinical evaluation Lameness evaluation	4 mo Group 1: return to racing 20 mo Group 1: no injuries, no lameness, high performance level	[39]

*Clinical trial*: 10 pz (46-69 yrs old) partially edentulous requiring bilateral or unilateral dental implants Autologous SVF (10^7^ nucleated cells) *β*TCP scaffold BCP (HA/*β*TCP 60%/40%) scaffold	Group 1: *β*TCP Group 2: *β*TCP+SVF Group 3: BCP Group 4: BCP+SVF	Histology Histomorphometry	6 mo All groups: =bone formation, blood vessels	[40]

*Clinical trial*: 10 pz (46-69 yrs) edentulous in the posterior maxilla Autologous SVF from abdomen *β*TCP scaffold BCP (HA/*β*TCP 60%/40%) scaffold	Group 1: *β*TCP Group 2: *β*TCP+SVF (20 × 10^6^) Group 3: BCP Group 4: BCP + SVF (10 × 10^6^)	Micro-CT Histomorphometry	6 mo Groups 2 and 4: ↑ BV/TV, OV/TV than groups 1 and 3 ≥36 mo No adverse effects	[41]

*Clinical trial*: 8 pz (62-84 yrs) with displaced low-energy fractures of the proximal humerus Autologous SVF from abdomen Fibrin hydrogel+porous silicate HA microgranules scaffold *In vivo*: Adult nude rats with a critically sized segmental defect in femora (6 mm) Fibrin hydrogel+porous silicate HA microgranules scaffold hSVF (8 × 10^6^) from abdomen	*Clinical trial*: Group 1: scaffold+SVF pelleted *In vivo*: Group 1: scaffold Group 2: scaffold+SVF	*Clinical trial*: Safety Histology Micro-CT *In vivo*: Micro-CT Histology	Clinical trial: 12 mo Group 1: no adverse reactions; ↓ pain during time; ↑ formed bone ossicles during time In vivo: 2 mo Group 2: ↑ mineralized volume, BV/TV, newly formed bone, frank bone, early stage of maturation than group 1	[42]

**Table 2:** *In vitro*, *in vivo*, and clinical studies on SVF in cartilage regeneration.

Experimental model	Treatment groups	Evaluations	Results	Ref.
*In vitro*: Human chondrocytes (2 × 10^5^) from healthy donors hSVF or hADSCs (2 × 10^5^) from abdomen *In vivo*: 10 BALB/C nude mice (6 wks) with subcutaneous pouches Allogenic SVF or ADSCs Allogenic chondrocytes (1 × 10^7^)	*In vitro*: Group 1: SVF pellet Group 2: ADSC pellet Group 3: chondrocyte pellet Group 4: SVF+chondrocyte pellet (4 : 1) Group 5: ADSCs+chondrocyte pellet (4 : 1) *In vivo*: Group 1: no treatment Group 2: SVF+chondrocytes (4 : 1) in 2% alginate Group 3: ADSCs+chondrocytes (4 : 1) in 2% alginate Group 4: chondrocytes	*In vitro*: Histology GAG quantification Cell proliferation *In vivo*: Histology GAG quantification IHC (COLL II)	In vitro: 1 mo Group 4: ↑ GAG, chondrocytes % than group 5 In vivo: 2 mo Group 2: ↑ GAG, COLL II protein than groups 1, 3, and 4	[43]

*In vivo*: 8 skeletally mature Dutch milk goats (82.4 ± 11.7 kg) with 2 osteochondral defects in the right troclea femoris and 2 defects in the right medial femoral condyles Autologous SVF or ADSCs (5 × 10^6^) COLL I/COLL III scaffold (5 × 3 mm)	Group 1: scaffold Group 2: scaffold+SVF Group 3: scaffold+ADSCs	Gross evaluations Histology Micro-CT	1 mo Group 1: =gross appearance score, histological score than groups 2 and 3. 4 mo Group 2: ↑ cartilage and SB regeneration than groups 1 and 3 Group 2: ↑ hyaline cartilage; ↓ fibrocartilage, cartilaginous tissue in SB than group 3	[44]

*In vivo*: 30 small tail Han sheep (6 mo) with OA in the right knee Autologous SVF (1 × 10^7^) or ADSCs from cervicothoracic region HA scaffold (600–1500 kDa) (2.5 ml)	Group 1: saline (5 ml) Group 2: scaffold Group 3: scaffold+ADSCs (1 × 10^7^) Group 4: scaffold+ADSCs (5 × 10^7^) Group 5: scaffold+SVF	IHC (COLL X, SDF1)	3 mo Group 5: ↓ COLL X protein than groups 3 and 4. Group 5: ↑ SDF1 protein than groups 1 and 2	[7]

*In vivo*: 9 NOD/SCID mice with injured cartilage hSVF (2 × 10^6^) from abdomen	Group 1: PBS Group 2: SVF+PRP	Movement recording Histology Histomorphometry	45 days Group 2: ↓ time required that mice could move, cartilage lesion area; ↑ formed neocartilage than group 1	[45]

*Clinical trial*: 13 pz (65-82 yrs) with bilateral severe OA Allogenic SVF (3 × 10^7^) from abdomen	Group 1: SVF	Clinical evaluation	1 and 6 mo Group 1: ↓ JKOM score, WOMAC score, VAS score than presurgery. No differences between 1 and 6 mo	[46]

*Clinical trial*: 18 pz (59.6 ± 10.5 yrs) with OA Autologous SVF (35 ml) from abdomen	Group 1: SVF	Clinical evaluation Radiography	3, 6, and 18 mo Group 1: ↓ VAS score, WOMAC % than presurgery. No infection, thromboembolism, adverse reaction 18 mo Group 1: =outerbridge score than presurgery	[47]

*Clinical trial*: 10 pz (≥50 yrs) with idiopathic knee OA Allogenic SVF (5 ml) from abdomen	Group 1: SVF+PRP (3 ml)	Clinical evaluation SF analysis	3, 6, 12, 18, and 24 mo Group 1: ↓ WOMAC total, WOMAC stiffness, WOMAC pain, WOMAC physical function; ↑ six-minute walk distance than presurgery 24 mo Group 1: no atypical cells in SF, restore SF properties, synovial metabolism; ↓ cartilage pathology than presurgery	[48]

*Clinical trial*: 4 pz (23-74 yrs) with OA of both knees Autologous SVF (5 × 10^7^, 6 × 10^7^, 7.5 × 10^7^, and 10 × 10^7^) from periumbilical region	Group 1: SVF+PRP (3 ml) (4 injections every month)	Clinical evaluation	12 mo Group 1: ↑ KOOS score pain, symptoms, ADL, sport/rec function, knee-related QOL. Pz regained normal functional activity	[49]

*Clinical trial*: 30 pz (>18 yrs) with OA Autologous SVF from abdomen	Group 1: arthroscopic microfracture Group 2: arthrosopic microfracture+SVF+PRP (5 ml)	Clinical evaluation BM edema	12 mo Group 2: ↓ BM edema than group 1 Group 1: ↓ WOMAC score during time 18 mo Group 2: ↓ WOMAC score during time Group 2: ↓ WOMAC score; ↑ Lysholm score than group 1	[50]

*Clinical trial*: 33 pz (>38 yrs) with OA Autologous SVF (6 ml with 90‐120 × 10^6^ cells) from abdomen	Group 1: arthroscopic microfracture Group 2: arthroscopic microfracture+SVF	Clinical evaluation BM edema	12 mo Groups 1 and 2: ↓ VAS score, WOMAC score during time 24 mo Group 2: ↓ VAS score, WOMAC score, Outerbridge score than group 1 Groups 1 and 2: ↑ Lysholm score during time Group 2: ↓ BM edema during time Group 1: ↑ BM edema during time	[51]

*Clinical trial*: 16 pz (53 ± 10.97 yrs) with bilateral OA of grade II or III Hyaluronic acid (4 ml) Autologous SVF (4 ml) from abdomen	Group 1: arthroscopic debridement+SVF Group 2: arthroscopic debridement+HA	Clinical evaluation Radiography	1, 3, 6, and 12 mo Group 1: ↓ VAS, WOMAC pain, WOMAC stiffness; ↑ ROM during time Group 2: ↑ VAS, WOMAC pain, WOMAC stiffness; ↓ ROM than group 1 1 mo Group 2: ↑ ROM during time 3, 6, and 12 mo Group 2: ↓ ROM during time 1 and 3 mo Group 2: ↓ VAS during time 6 and 12 mo Group 2: ↑ VAS during time Group 1: ↑ MOCART score during time Group 1: ↑ MOCART score, complete tissue filling than group 2 Group 2: ↓ MOCART score during time	[52]

*Clinical trial*: 26 pz (46.1 ± 12.2 yrs) with full-thickness chondral defect of the talar dome Autologous SVF (3.94 × 10^6^ MSCs) from buttock	Group 1: arthroscopic marrow stimulation Group 2: arthroscopic marrow stimulation+SVF	Clinical evaluation Radiography	16-25 mo Groups 1 and 2: ↓ VAS score; ↑ AOFAS score during time Group 2: ↓ VAS score; ↑ AOFAS score, Tegner score, MOCART score than group 2	[53]

*Clinical trial*: 1 pz (36 yrs) with traumatic osteochondral lesion of the right medial femoral condyle 8 mo after injury Autologous SVF (1.5 × 10^6^) from abdomen	Group 1: microfracture+SVF	Clinical evaluation BM edema Radiography	24 mo Group 1: ↑ IKDC, EQ-VAS than presurgery 12 and 24 mo Group 1: recovery of CT; ↓ BM edema than presurgery	[54]

**Table 3:** *In vitro*, *in vivo*, and clinical studies on SVF in tendon regeneration.

Experimental model	Treatment groups	Evaluations	Results	Ref
*In vivo*: 20 adult NZW rabbits (2.5-3 kg) with complete transaction of DDFT midsubstance Allogenic SVF (4 × 10^6^) from inguinal region	Group 1: PBS (0.2 ml) Group 2: SVF	Histology Histomorphometry IHC (COLL I, COLL III) Biomechanics	2 mo Group 2: ↑ fibrillar linearity, fibrillar continuity, COLL I protein, ultimate load, energy absorption, stiffness; ↓ no. of capillaries in neotendon, COLL III protein than group 1	[55]

*In vivo*: 36 adult NZW rabbits (2.5-3 kg) with a complete transaction through the central one third of flexor tendon Allogenic SVF (4 × 10^6^) from inguinal region Allogenic BMSCs (4 × 10^6^) from iliac crest	Group 1: PBS (0.2 ml) Group 2: SVF Group 3: BMSCs	Biomechanics	3 and 8 wks Groups 2 and 3: ↑ energy absorption, ultimate load, ultimate stress, yield load, stiffness than group 1	[56]

*In vivo*: 36 adult NZW rabbits (2-2.5 kg) with supraspinatus tendon severed from the great trochanter Autologous SVF from inguinal region	Group 1: FG (1 ml) Group 2: SVF+FG (1 ml)	Radiography Biomechanics	1 mo Group 2: ↑ maximum load, maximum strength than group 1 2 mo Group 2: ↑ maximum load, maximum strength, stiffness than group 1 3 mo Group 2: ↑ SNQ, stiffness than group 1	[57]

*In vivo*: 36 NZW rabbits (2-2.5 kg) with bilateral supraspinatus tendon severed from the great trochanter Autologous SVF from inguinal region	Group 1: FG (1 ml) Group 2: SVF+FG (1 ml)	Histology IHC (COLL I, COLL III, BMP2) Histomorphometry Biomechanics	2 mo Group 2: ↑ tendon-bone healing maturity, COLL I, COLL III, COLL I/COLL III, BMP2 protein, maximum load, maximum strength, stiffness than group 1	[58]

*Clinical trial*: 43 pz (29-55 yrs) with unilateral or bilateral NIAT Allogenic SVF (4 ml) from abdomen	Group 1: PRP (4 ml) Group 2: SVF	Radiography Clinical evaluation	6 mo Groups 1 and 2: ↓ VAS; ↑ MR size, US size, PD than presurgery Group 1: ↓ MR-Si than group 2	[59]

*Clinical trial*: 44 pz (18-55 yrs) with unilateral or bilateral chronic tendinopathy of the Achilles tendon Allogenic SVF (4 ml) from abdomen	Group 1: PRP (4 ml) Group 2: SVF	Clinical evaluation	2 wks, 1 mo, 4 mo, and 6 mo Groups 1 and 2: ↓ VAS pain scale; ↑ VISA-A score, AOFAS score, SF-36 than presurgery 2 wks Group 2: ↓ VAS pain scale; ↑ VISA-A score, AOFAS score than group 1 1 mo Group 2: ↓ VAS pain scale than group 1	[60]

**Table 4:** *In vitro*, *in vivo*, and clinical studies on AECs in bone regeneration.

Experimental model	Treatment groups	Evaluations	Results	Ref
*In vitro*: hAECs Purchased hADSCs	Group 1: hAECs (2.1 × 10^4^) cultured in OM Group 2: hADSCs (2.1 × 10^4^) cultured in OM Group 3: hAECs (1 × 10^5^ per pellet) cultured in CM Group 4: hADSCs (1 × 10^5^ per pellet) cultured in CM	RT-PCR (RUNX2, SOX9) Alizarin red staining Alcian blue staining IHC (OCN, AGG, and COLL II)	3, 7, and 14 days Group 1: ↑ RUNX2 gene expression, OCN protein than group 2 Group 3:↑ AGG and COLL II proteins, SOX9 gene expression, than group 4 2 and 4 wks Group 1: ↑ mineralization during time	[61]

*In vitro*: hAECs PEMF = 50 Hz, 1 mT for 30 min each time, 2 for days with an interval of 12 hours	Group 1: hAECs in NM Group 2: hAECs in OM Group 3: hAECs in NM+PEMF Group 4: hAECs in OM+PEMF	RT-PCR (ALP, OCN, BMP2, RUNX2, NRF2, and KEAP1) ALP activity IHC (OCN) Calcium deposition	3, 7, and 11 days Group 4: ↑ ALP, OCN gene expression than groups 1-3 Group 3: ↑ ALP, OCN than groups 1 and 2 Group 2: ↑ ALP, OCN than group 1 7 days Group 3: ↑ ALP activity, calcium deposition than group 1 7, 11, and 21 days Group 2: ↑ ALP activity than groups 1 and 3 Group 4: ↑ ALP activity, OCN protein, calcium deposition than groups 1-3 Group 3: ↑ OCN protein than group 1 21 days Group 2: ↑ OCN protein, calcium deposition than groups 1 and 3 3, 11, and 21 days Group 4: ↑ BMP2, RUNX2, NRF2, and KEAP1 gene expression than groups 1-3	[62]

*In vitro*: Conditioned medium from hAECs after 24 hrs of culture Purchased hFOB1.19 (5 × 10^3^)	Group 1: hFOB1.19 in NM Group 2: hFOB1.19 in conditioned medium Group 3: hFOB1.19 in conditioned medium+TGF*β*1 antibody (5 *μ*g/ml)	Cell proliferation Cell migration ALP activity RT-PCR (ALP, OCN, OPN, and RUNX2)	2 hrs Group 2: ↑ hFOB1.19 migration than group 1 1, 2, and 3 days Group 2: ↑ hFOB1.19 proliferation than group 1 6 days Group 2: ↑ hFOB1.19 ALP activity, ALP, OCN, OPN, RUNX2 gene expression than group 1 Group 3: ↓ hFOB1.19 ALP activity, ALP, OCN gene expression than group 2 6 hrs Group 3: ↓ hFOB1.19 migration than group 2	[63]

*In vitro*: hAECs (3rd-5th passage) from 6 patients (32 ± 4.5 yrs)	Group 1: hAECs in NM Group 2: hAECs+mechanical stretch (maximum uniaxial stretched length (7.35 cm) for 2, 6, 12, and 24 hrs with 5% elongation at a frequency of 0.5 Hz Group 3: hAECs in OM for 21 days Group 4: hAECs+OM for 21 days+mechanical stretch for 2, 6, 12, and 24 hrs Group 5: hAECs+Runx2 shRNA+OM for 24 hrs Group 6: hAECs+Runx2 shRNA+mechanical stretch for 24 hrs Group 7: hAECs+Runx2 shRNA+OM+mechanical stretch for 24 hrs	RT-PCR (ALP, OCN, RUNX2, *β*-catenin, and Cyclin D1) WB (ALP, OCN, RUNX2, *β*-catenin, and Cyclin D1)	12 hrs Groups 3 and 4: ↑ OCN protein than group 1 6, 12, and 24 hrs Group 3: ↑ RUNX2, ALP, and OCN gene expression than group 1 6 and 12 hrs Group 4: ↑ RUNX2, ALP, OCN gene expression and protein than groups 2 and 3 12 hrs Group 3: ↑ *β*-catenin, RUNX2, Cyclin D1 gene expression and protein than group 1 Group 4: ↑ *β*-catenin, RUNX2, Cyclin D1 gene expression and protein than groups 2 and 3 24 hrs Groups 5-7: ↓ RUNX2, OCN gene expression, and protein than groups 2-4	[64]

*In vivo*: 6 adult sheep (2 yrs) that need bilateral sinus augmentation Allogenic oAECs (1 × 10^6^) from 3 slaughtered sheep (3 mo of pregnancy) CaP (HA-*β*TCP: 30/60) scaffold	Group 1: scaffold Group 2: scaffold+AECs	Micro-CT Histology Histomorphometry IHC (VEGF)	45 days Group 2: ↑ VEGF, VA; ↑ BA than group 1 3 mo Group 2: ↓ VEGF, VA; ↑ BA than group 1	[65]

*In vivo*: 16 SD rats (7 wks) with unilateral maxillary alveolar defect hAECs *β*TCP scaffold (4 × 3 × 3 mm)	Group 1: scaffold Group 2: scaffold+AECs	Micro-CT Histology Histomorphometry IHC (CD68, VEGF)	1 mo Group 2: ↓ Tb.Sp, CD68 than group 1 2 mo Group 2: ↑ BMD, BV/TV, Tb.N, BA, VEGF area, CD68 than group 1	[66]

*In vivo*: 3 nude mice with subcutaneous pouches hAECs, hBMSCs, hAFMSCs *β*TCP scaffold (5 × 10 mm)	Group 1: scaffold Group 2: scaffold+hAEC Group 3: scaffold+hBMSCs Group 4: scaffold+hAFMSCs	Histology IHC (OPN, OCN)	1 mo All groups: no well-mineralized islands Groups 2-4: viable cells; ↑ OPN, OCN proteins than group 1	[67]

**Table 5:** *In vitro*, *in vivo*, and clinical studies on AECs in cartilage regeneration.

Experimental model	Treatment groups	Evaluations	Results	Ref
*In vitro*: Cartilage samples with defect of 2 mm Human chondrocytes from femoral heads or knee (1 × 10^6^) hBMSCs from femoral heads of donors (50-70 yrs) hAECs hAMSC HAM scaffold	Group 1: scaffold seeded with chondrocytes+chondrocyte pellet (6 × 10^5^) Group 2: scaffold seeded with BMSCs+BMSC pellet (6 × 10^5^) Group 3: scaffold seeded with AECs+AEC pellet (6 × 10^5^) Group 4: scaffold seeded with AMSCs+AMSC pellet (6 × 10^5^)	Histology IHC (COLL I, COLL II)	2 mo Group 1: =repair of lesioned area, ICRS score than groups 2, 3, and 4 Group 4: ↑ COLL II protein than group 1 Group 2: ↑ COLL I protein than groups 1, 3, and 4	[68]

*In vitro*: hAECs in micromasses (1 × 10^7^)	Group 1: micromasses+TGF*β*1 (1 ng/ml) Group 2: micromasses+BMP7 (100 ng/ml)	RT-PCR (SOX9, COLL II, PG)	3 days Group 2: ↑ SOX9, COLL II gene expression than group 1 3 wks Group 1: ↑ SOX9, COLL II gene expression than group 2 Groups 1 and 2: ↑ PG gene expression during time	[69]

**Table 6:** *In vitro*, *in vivo*, and clinical studies on AECs in tendon regeneration.

Experimental model	Treatment groups	Evaluations	Results	Ref
*In vitro*: oAECs (1 × 10^4^) Tenocytes (1 × 10^4^) or tendons from heel of adult sheep (2-3 yrs) or fetus sheep (2-3 mo)	Group 1: AECs Group 2: AECs+fetal tenocytes Group 3: AECs+adult tenocytes Group 4: AECs+fetal tendons Group 5: AECs+adult tendons	AEC proliferation AEC migration AEC telomere analysis RT-PCR (COLL I, COLL III) IHC (OCN, TNMD, and SCXB)	7 days Group 4: ↑ AEC migration than group 1 2 wks Groups 3 and 5: ↓ AEC proliferation than group 1 1 mo Groups 2 and 4: ↑ AEC proliferation, OCN, TNMD, and SCXB proteins than group 1 Group 4: ↓ TEA, TEF, and MEAND than group 1 Groups 2-5: ↑ COLL I gene expression than group 1 Group 3: ↓ COLL III gene expression than group 1 Groups 3 and 5: ↓ TNMD protein than group 1	[70]

*In vivo*: 15 horses with acute SDFT monolateral injuries oAECs (7 × 10^6^) from slaughtered sheep at 3 mo of pregnancy	Group 1: healthy tendons Group 2: AECs	Histology IHC (COLL III, COLL I, and Ki-67)	2 mo Group 2: ↓ COLL III protein; ↑ COLL I, Ki-67 proteins than group 1, no immunological reaction	[71]

*In vivo*: 6 adult horses (3-7 yrs) with acute or chronic SDFT tendinopathy in the midmetacarpal region; oAECs (7 × 10^6^) from slaughtered sheep at 3 months of pregnancy	Group 1: AECs	Ultrasonography Histology	6 mo Group 1: cross-sectional area similar to an healthy tendon, collagen fibers parallel to the longitudinal axis of the tendon, no neovascularization than presurgery	[72]

*In vivo*: 23 sheep (40 kg) with a bilateral full-thickness FDST hole (3 mm diameter) Allogenic oAECs (4 × 10^6^) from slaughtered sheep at 3 months of pregnancy	Group 1: FG Group 2: AECs+FG	Histology RT-PCR (COLL I, COLL III) Histomorphometry Biomechanics	2 wks Group 2: ↑ tendon microarchitecture than group 1 1 mo Group 2: ↑ tendon microarchitecture, proliferation, COLL I gene expression, maximum failure load, stiffness; ↓ VA, leukocyte infiltration, cellularity, COLL III gene expression than group 1	[73]

*In vivo*: 3 adult sheep (50 kg) with bilateral defect in the Achilles tendon middle portion Allogenic oAECs (2 × 10^6^) from slaughtered sheep at 60-80 days of pregnancy	Group 1: no treatment Group 2: AECs	Histology	1 mo Group 2: absent inflammation-proliferating cells, collagen fibers started to be oriented along a longitudinal axis Group 1: no tendon healing, no proliferating cells, nonorganized collagen fibers	[74]

*In vivo*: 18 Appenninica breed sheep (2 yrs) with a full-thickness hole in FDST (3 mm diameter) Allogenic oAECs (4 × 10^6^)	Group 1: FG Group 2: FG+oAECs	Histology IHC (COLL I, CD45, CD68, CD86, and MMR/CD206) RT-PCR (CD86, CD206, YM1, IL10, and IL12b)	7, 14, and 28 days Group 2: ↑ tissue organization; ↓ leukocyte infiltration, IL12 b gene expression than group 1 14 and 28 days Group 2: ↓ macrophage infiltration, CD86 gene expression, CD86 positive cells; ↑ IL10 gene expression than group 1 28 days Group 2: ↑ COLL I; ↓ cellularity, YM1 gene expression, CD206-positive cells than group 1	[75]

*In vivo*: 29 adult sheep (2 yrs) with bilateral Achilles tendon defects (5 mm diameter) hAECs (1 × 10^7^)	Group 1: FG Group 2: FG+hAECs	RT-PCR (COLL I, COLL III, TNMD, THBS4, CD206, IL10, VEGF, CD68, IL12b) IHC (COLL I, COLL III, VEGF) Histology Biomechanics (maximum load, stiffness)	1 mo Group 2: rapid recovery of the tissue; sporadic edema; regular longitudinal profile; ↑ maximum failure load; stiffness; regularly arranged fibers; COLL I gene expression and protein, TNMD, THBS4, CD206, and IL10 gene expression; ↓ COLL III and VEGF gene expression and protein, CD86, and IL12b gene expression than group 1	[76]

Abbreviations: hSVF = human stromal vascular fraction; hBMSCs = human bone marrow mesenchymal stem cells; yrs = years; hrs = hours; wks = weeks; BMSCs = bone marrow mesenchymal stem cells; SVF = stromal vascular fraction; ALP = alkaline phosphatase; RT-PCR = reverse transcriptase-polymerase chain reaction; RUNX2 = runt-related transcription factor 2; COLL = collagen; OSX = Osterix; hADSCs = human adipose-derived mesenchymal stem cells; ADSCs = adipose-derived mesenchymal stem cells; mm = millimeters; BA/TA = bone area/tissue area; PLCL = poly(L-lactide-co-caprolactone); NM = normal medium; CM = chondrogenic medium; OM = osteogenic medium; SOX = sex-determining region Y boxes; OCN = osteocalcin; ACAN = aggrecan; mo = months; IHC = immunohistochemistry; Micro-CT = microcomputed tomography; HA = hydroxyapatite; cm = centimeters; BV/TV = bone volume/tissue volume; CD = cluster of differentiation; *β*TCP = beta-tricalcium phosphate; CFU = colony forming units; ml = milliliters; BA = bone area; OPN = osteopontin; PRP = platelet-rich plasma; mg = milligrams; PCL-DCB = polycaprolactone-decellularized bovine bone extracellular matrix; BV = bone volume; VA = vascular area; PLA = polylactic acid; DBM = demineralized bone matrix; SD = Sprague-Dawley; BVF = bone volume fraction; Tb.Th = trabecular thickness; PLGA = poly(lactide-co-glycolide); NZW = New Zealand White; BMD = bone mineral density; Tb.Sp = trabecular separation; PMN = polymorphonuclear; ONJ = osteonecrosis of the jaw; pz = patients; BCP = biphasic calcium phosphate; OV/TV = osteoid volume/tissue volume; GAG = glycosaminoglycans; kg = kilograms; SB = subchondral bone; OA = osteoarthritis; kDa = kilodaltons; SDF = stromal cell-derived factor; PBS = phosphate-buffered solution; NOD/SCID = nonobese diabetic/severe combined immunodeficiency; JKOM score = Japanese Knee Osteoarthritis Measure; VAS score = visual analog scale score; WOMAC score = the Western Ontario and McMaster Universities Osteoarthritis score; SF = synovial fluid; KOOS score = Knee Injury and Osteoarthritis Outcome Score; ADL = Activities of Daily Living; QOL = quality of life; BM = bone marrow; ROM = range of motion; MOCART = magnetic resonance observation of cartilage repair tissue; MSCs = mesenchymal stem cells; AOFAS score = American Orthopedic Foot and Ankle Score; IKDC = International Knee Documentation Committee; EQ-VAS = EuroQol-visual analog scale; CT = cartilage thickness; DDFT = deep digital flexor tendon; no. = number; FG = fibrin glue; SNQ = signal-to-noise quotient; BMP = bone morphogenetic protein; NIAT = noninsertional Achilles tendinopathy; PD = peri- and intratendinous flow; MR-Si = MR signal intensity; VISA-A score = Victorian Institute of Sports Assessment—Achilles questionnaire; SF-36 = Short Form 36 Health Survey; AECs = amniotic endothelial cells; hAECs = human amniotic endothelial cells; NRF2 = nuclear factor erythroid 2-related factor 2; KEAP1 = Kelch-like ECH-associated protein 1; PEMF = pulsed electromagnetic field; Hz = Hertz; min = minutes; hFOB = human fetal osteoblastic; TGF*β*1 = transforming growth factor *β*1; *μ*g = micrograms; VEGF = vascular endothelial growth factor; oAECs = ovine AECs; CaP = calcium phosphate; Tb.N = trabecular number; hAFMSCs = human amniotic fluid mesenchymal stem cells; hAMSCs = human amniotic membrane mesenchymal stem cells; HAM = human amniotic membrane; ICRS score = International Cartilage Repair Society; PG = polygalacturonase; TNMD = tenomodulin; SCXB = scleraxis; TEA = telomere area; TEF = feret maximum; MEAND = mean densitometric value; SDFT = superficial digital flexor tendon; FDST = flexor digitorum superficialis tendon.

## Data Availability

The data supporting this systematic review are from previously reported studies and datasets, which have been cited.
